# Chronic Interstitial Nephritis in Childhood

**Published:** 1897-06

**Authors:** 


### CHRONIC INTERSTITIAL NEPHRITIS IN CHILDHOOD

Nephritis occurring during the years of childhood is so commonly consequent on and due to the acute infectious and exanthematous diseases, and it is so frequently of the exudative and parenchymatous varieties, that we are sometimes inclined to forget that interstitial nephritis is occasionally met with even in patients of the tenderest age. The truth of this must be apparent to the readers of a carefully prepared article on this subject, which has appeared in the last few numbers of the *Lancet* from the pen of Dr. L. G. Guthrie. This writer considers in great detail the diagnosis, clinical history, prognosis, etiology and treatment, basing his remarks largely on the clinical and autopsical histories of seven cases, one of which was a personal observation. The writer holds to the view that granular kidney is not the product of parenchymatous atrophy, but of interstitial inflammation, consisting of a round cell infiltration of the renal stroma, beginning in the cartex and spreading in the form of bands toward the center of the organ, notwithstanding the fact that the occurrence of contracted kidney in children has been used as an argument in favor that the contraction is in reality dependent upon a post-parenchymatous atrophy.

The diagnosis oftentimes presents no difficulty, and especially, if the cutaneous, gastro-intestinal, vascular, urinary and nervous accompaniments, which are in their association considered almost pathognomonic, are present. On the other hand, when one or several of these conditions are absent, the diagnosis can only be conjectured. The most unvarying accompaniment of interstitial nephritis in children is cardio-vascular hypertrophy and high arterial tension, and these, in connection with the well-known urinary and nervous symptoms, constitute the symptomatology of the disease. The author points out that children suffering from interstitial nephritis rarely develop edema, and when it does occur, it is with scanty, smoky and albuminous urine, and the condition of the skin and vascular systems will serve to distinguish it from ordinary acute nephritis. It is well to note that the author is in accord with other writers on this subject, in saying that the absence of albumin, even after frequent and systematic testing, does no exclude the possible existence of the disease. The writer lays becoming stress upon persistent gastro-intestinal symptoms, violent periodic headaches, associated with vomiting or vertigo, and convulsions. These symptoms have the same significance occurring in a child, as in an adult, and the necessity of excluding other diseases, such as cerebral tumor, migranous and uric acid headaches, attended with a similar symptomatology, is just as urgent. All in all, the most reliable symptom in excluding such conditions is the objective one of the cardio-vascular hypertrophy and high arterial tension. This condition absolutely never occurs in the former disease, in fact, directly the opposite state of affairs exists; while for the latter, the therapeutic test, and the presence of uric acid and oxalate crystals in the urine generally suffices to make the diagnosis. Finally, the writer concludes, the symptoms of interstitial nephritis are as insidious and gradual as the morbid process itself. In many cases only the existence of the disease can be suspected.

The prognosis of interstitial nephritis in children stands in direct relation to the cardio-vascular hypertrophy, the effects of the latter being to avert the accidents, or compensate for the shortcomings of the renal disease. Indications of excessive or of failing cardio-vascular hypertrophy are alike of bad omen, and the object of treatment should be to maintain the happy medium, and, to fulfill this end, the treatment is very largely symptomatic, not curative. When cardio-vascular hypertrophy is carried to excess, the arterial tension and heart must be relieved by the administration of vascular depressants, the nitrites, and the nitrates. These act by facilitating the elimination of effete products by all the emunctories, the cutaneous, internal and urinary systems. When, on the other hand, the cardio-vascular hypertrophy begins to show signs that it is no longer able to keep up the compensation, then the cardio-vascular excitants must be resorted to.

Although the writer shows that he is not unmindful of the fact that the life of these patients is generally proportionate in length to the supervision and care exercised in their hygiene, and to the prompt meeting of incidental symptoms as they arise, he is convinced that the guiding principle in the treatment should be "watch the heart and pulse."—*Pediatrics.*

